# Entero-Appendiceal Fistula Presenting with Overt Gastrointestinal Bleeding: A Case Report

**DOI:** 10.70352/scrj.cr.26-0299

**Published:** 2026-08-27

**Authors:** Seiya Sato, Taro Munechika, Ryohei Nomaru, Keiichi Shiokawa, Issei Takeshita, Kurumi Sahara, Nobuaki Kuno, Yoshiko Matsumoto, Ken Nagata, Hideki Nagano, Hiroyuki Takahashi, Gumpei Yoshimatsu, Suguru Hasegawa

**Affiliations:** 1 Department of Gastroenterological Surgery, Fukuoka University Hospital, Fukuoka, Fukuoka, Japan; 2 Department of Gastroenterology, Fukuoka University Hospital, Fukuoka, Fukuoka, Japan

**Keywords:** entero-appendiceal fistula, hematochezia, small bowel bleeding, double-balloon enteroscopy, laparoscopic small bowel resection

## Abstract

**INTRODUCTION:**

Entero-appendiceal fistula is an extremely rare entity, with very few cases reported in the literature. Most previously described cases presented with abdominal pain or were diagnosed incidentally. To our knowledge, there have been no reports of such a fistula presenting with overt gastrointestinal bleeding.

**CASE PRESENTATION:**

A 56-year-old man with a medical history of human immunodeficiency virus infection, alcoholic cirrhosis, and paroxysmal atrial fibrillation, who was under regular follow-up at our hospital, presented with a 4-day history of hematochezia that was occurring 3–4 times per day. He was found to have severe anemia with a hemoglobin level of 6.6 g/dL and was emergently hospitalized on the same day. Contrast-enhanced CT did not reveal active extravasation. Colonoscopy showed multiple diverticula but no clear bleeding source. Recurrent hematochezia prompted repeat imaging, and subsequent CT showed extravasation in the ileum. Colonoscopy again revealed blood originating from a segment proximal to the reach of the colonoscope, suggesting bleeding from a more proximal segment. Double-balloon enteroscopy revealed a hemorrhagic submucosal tumor-like lesion located approximately 100 cm proximal to the ileocecal valve. Biopsy was inconclusive, but surgical resection was indicated because of the ongoing bleeding and the need to exclude malignancy. Laparoscopic resection revealed that the tip of the appendix had formed a fistula into the ileal lumen. Histopathological examination confirmed a full-thickness fistula between the appendix and ileum, with no evidence of malignancy.

**CONCLUSIONS:**

This is the first reported case of an entero-appendiceal fistula presenting with overt gastrointestinal bleeding and endoscopic features mimicking a submucosal tumor. Although extremely rare, this condition should be considered in the differential diagnosis of small intestinal tumors, particularly in patients with a history of complicated appendicitis or immunosuppression.

## Abbreviations

AIDS
: acquired immune deficiency syndrome
CD4
: cluster of differentiation 4
HIV
: human immunodeficiency virus

## INTRODUCTION

Fistula formation between the appendix and small intestine is exceedingly rare. Appendiceal fistulas involving the bladder, rectum, and sigmoid colon have been reported more frequently, but only a few cases of entero-appendiceal fistulas have been documented.^1–3)^ These fistulas are often associated with appendiceal abscess, inflammation, or a neoplastic process and typically present with abdominal pain or are detected incidentally. Presentation with overt gastrointestinal bleeding and tumor-like features is exceptionally rare, and to our knowledge, no such cases have been previously documented. In this report, we describe a unique case in which such a fistula was identified during evaluation for overt gastrointestinal bleeding in a patient with a history of complicated appendicitis and HIV infection.

## CASE PRESENTATION

A 56-year-old man with a medical history of HIV/AIDS, alcoholic liver disease, paroxysmal atrial fibrillation, and complicated appendicitis that had been managed conservatively presented to our hospital with a 4-day history of hematochezia that was occurring 3–4 times daily. His hemoglobin level had decreased from 13.1 to 6.6 g/dL over the preceding month. On admission, his vital signs were stable, but he appeared pale and showed clinical signs of severe anemia, including conjunctival pallor. He was receiving apixaban for atrial fibrillation. Furthermore, although the patient had a history of alcoholic cirrhosis, the platelet count and coagulation parameters were within normal limits at presentation.

At the time of conservative treatment for perforated appendicitis, the patient’s CD4+ T-cell count was 30 cells/μL and the HIV RNA level was 1100000 copies/mL. Antiretroviral therapy (bictegravir/tenofovir alafenamide/emtricitabine) was subsequently initiated, but the CD4+ T-cell count remained below 250 cells/μL for more than a year. The current lesion was diagnosed 4 years after the onset of perforated appendicitis.

**Fig. 1:**
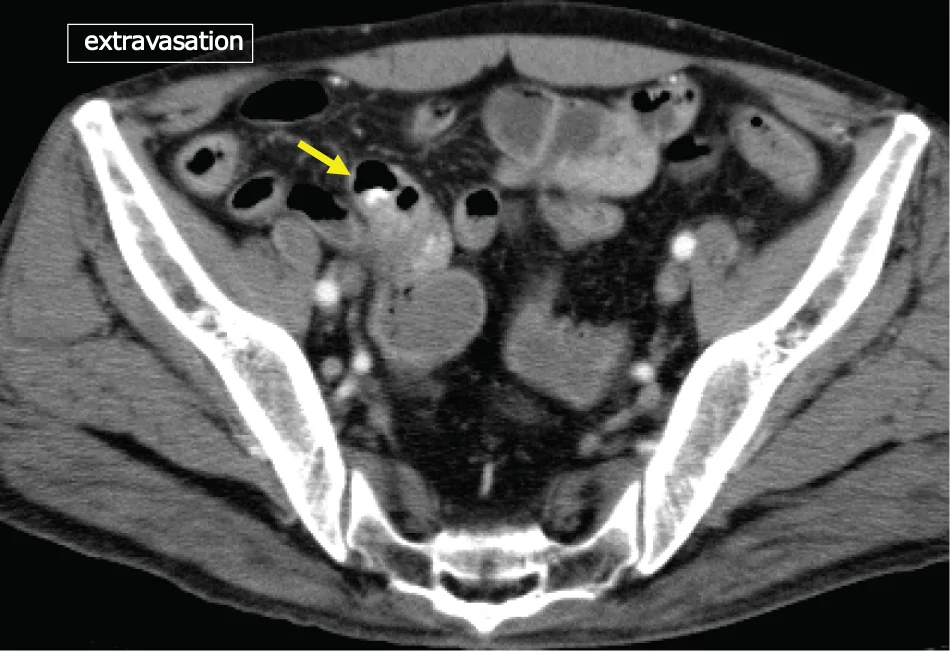
Contrast-enhanced CT findings. Contrast-enhanced CT demonstrated a hyperdense area in the colon and extravasation of contrast in the ileum, indicating active gastrointestinal bleeding without a clear mass lesion.

Initial contrast-enhanced abdominal CT revealed a hyperdense area in the colon and contrast extravasation in the ileum without any definite mass lesion (**Fig. 1**). Colonoscopy revealed multiple colonic diverticula with blood residue, but no clear bleeding source. Blood was observed in the terminal ileum, suggesting bleeding from a more proximal small bowel segment.

**Fig. 2:**
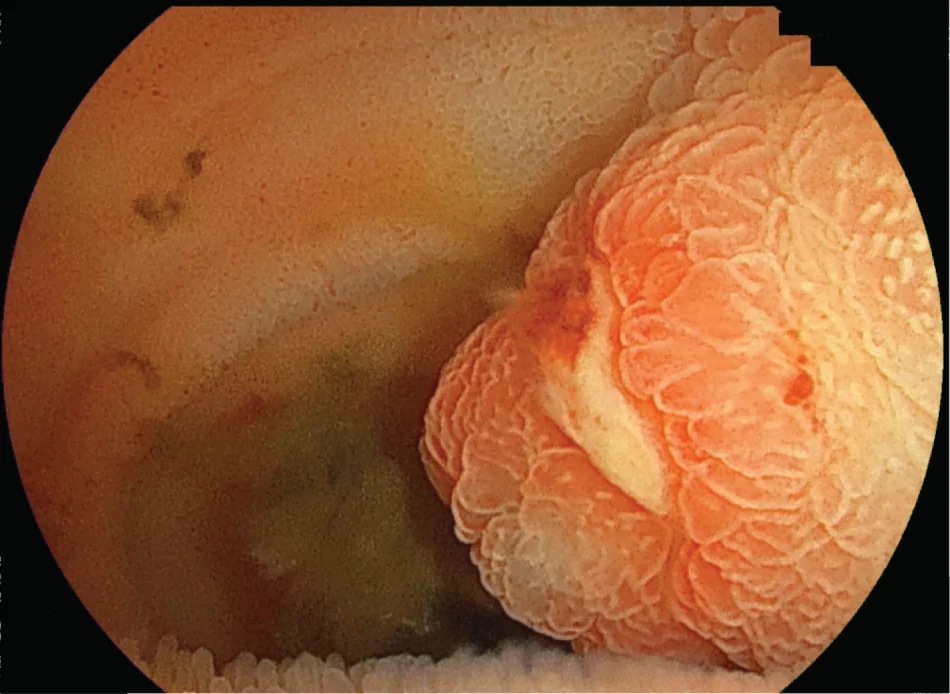
Colonoscopy findings. Colonoscopy revealed blood residue in the terminal ileum without identification of a definitive bleeding source in the colon. Subsequent double-balloon enteroscopy identified a hemorrhagic, ulcerated submucosal lesion located approximately 100 cm proximal to the ileocecal valve, mimicking a small intestinal tumor.

Double-balloon enteroscopy was performed on hospital day 3, revealing a submucosal tumor-like, hemorrhagic lesion approximately 100 cm proximal to the ileocecal valve. The lesion was ulcerated and bled easily upon contact (**Fig. 2**). Tattooing was performed, and biopsy revealed inflammatory exudate with atypical cells; however, the findings were inconclusive for malignancy.

**Fig. 3:**
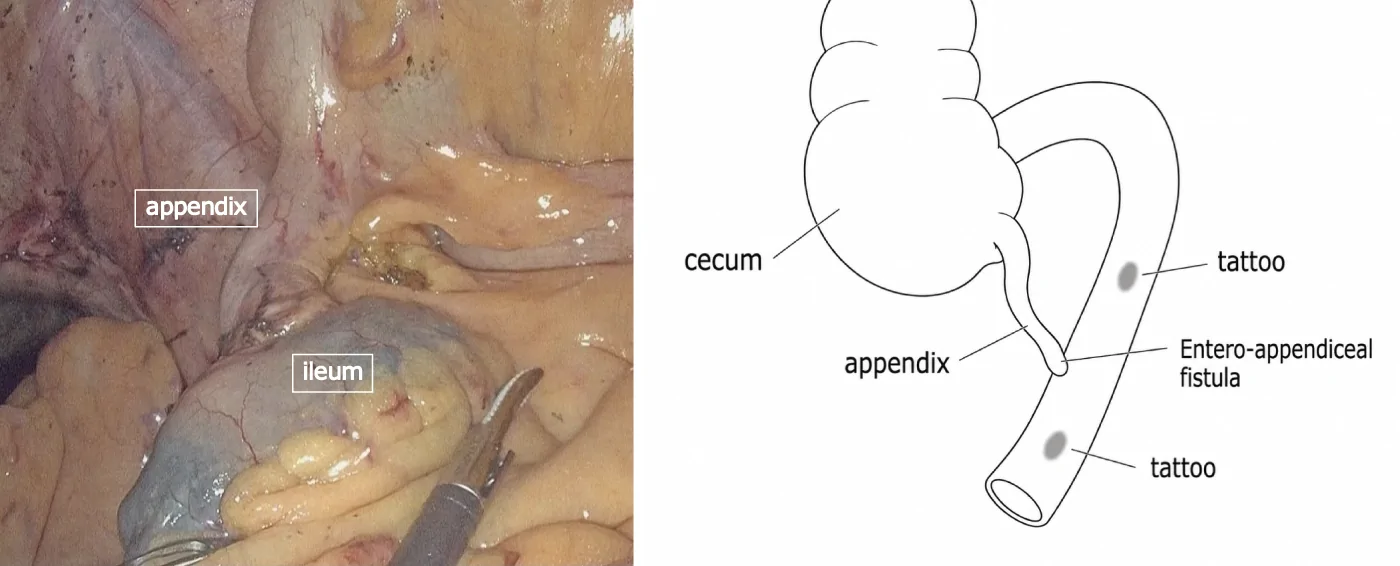
Intraoperative images. Laparoscopic exploration demonstrated dense adhesions involving 2 segments of the ileum, the appendix, and the right lower abdominal wall. The tattooed site corresponds to a segment adherent to the tip of the appendix.

Given the ongoing bleeding, uncertain pathology, and potential malignancy, laparoscopic small bowel resection was planned. Intraoperatively, adhesions were noted between 2 segments of the ileum, the appendix, and the right lower abdominal wall. The tattooed site corresponded to a segment adherent to the tip of the appendix (**Fig. 3**). Given the dense adhesions and the patient’s history of perforated appendicitis, en bloc resection of the ileum and appendix was performed. The lesion was not a typical epithelial tumor, and macroscopic findings were not suggestive of malignancy. Judging that the possibility of malignancy was low, a partial resection without lymph node dissection was performed to avoid excessive invasiveness.

**Fig. 4:**
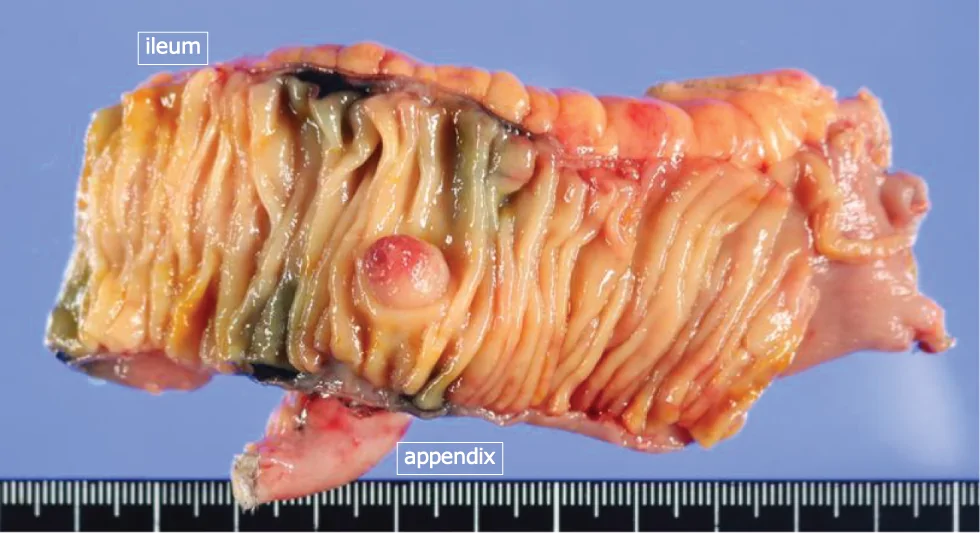
Gross appearance of the resected specimen. The tip of the appendix is seen protruding into the lumen of the ileum at the site of an ulcerated lesion in the submucosa. The fistulous tract between the appendix and ileum is evident on gross inspection.

Gross examination of the resected specimen revealed that the tip of the appendix protruded into the ileal lumen at the site of a submucosal-appearing lesion with ulceration (**Fig. 4**).

**Fig. 5:**
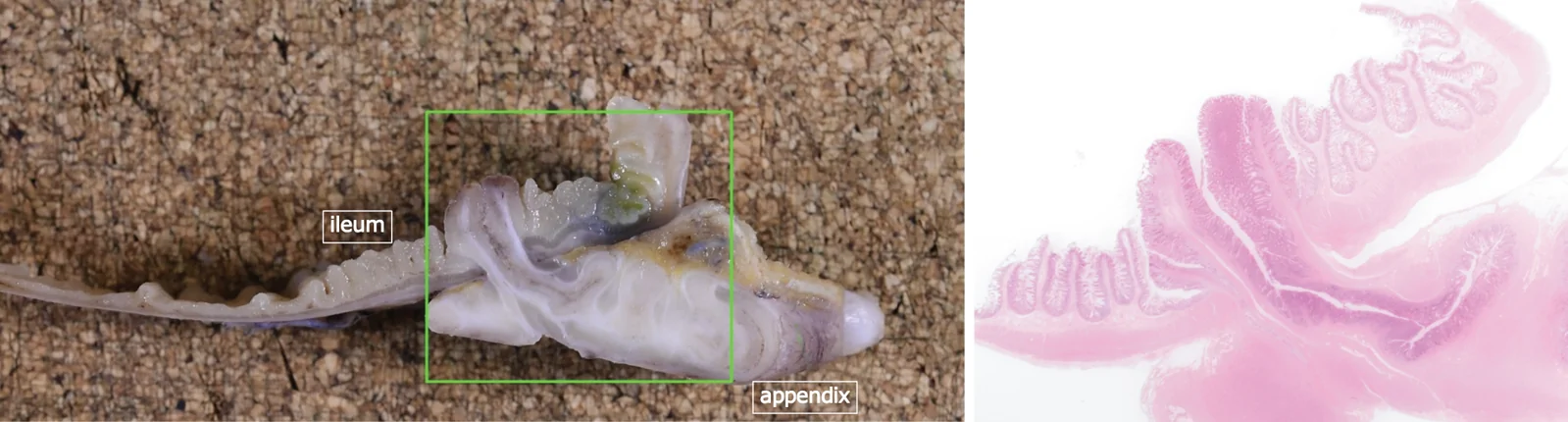
Histological findings of the entero-appendiceal fistula. Hematoxylin–eosin staining revealed full-thickness penetration of the appendiceal wall into the ileum. The mucosal layers of the appendix and ileum are continuous, with disruption of the muscularis propria at the fistula site. No evidence of malignancy was observed (original magnification 4×). The green box in the left panel indicates the area corresponding to the histological section shown in the right panel.

Histological analysis showed full-thickness penetration of the appendiceal wall into the ileum, with merging of the mucosa of the appendix and ileum. The muscularis propria of both structures was disrupted at the point of fistulization (**Fig. 5**). No evidence of malignancy was observed.

The patient had an uneventful postoperative course and was discharged home on POD 12. No recurrence of symptoms has been observed to date.

## DISCUSSION

Fistulas involving the appendix and other intra-abdominal organs are uncommon. Appendico-vesical and appendico-colonic fistulas are among the more frequently reported types, whereas entero-appendiceal fistulas remain exceedingly rare. A search of the PubMed database using the terms “appendix,” “small intestine,” and “fistula” yielded only 4 reported cases of such fistulas, with etiologies that included Meckel’s diverticulum, appendiceal diverticulum, congenital anomalies, and adhesive intestinal obstruction.^1,4–6)^

In the present case, the fistula originated approximately 100 cm proximal to the terminal ileum, which is a relatively mobile region of the small intestine. Fistula formation typically requires prolonged tissue apposition. Therefore, fistulas are more frequently observed between fixed intra-abdominal structures such as the stomach and duodenum. In contrast, fistulas involving more mobile segments of the small intestine, such as the jejunum or the mid-ileum, are rare and tend to occur in relatively fixed areas, such as within 50 cm of the ligament of Treitz or the terminal ileum.

Although there are no specific reports demonstrating an increased incidence of appendiceal fistula formation in HIV-positive patients, previous studies have shown that HIV-associated immunosuppression is associated with complicated appendicitis and a decrease in CD4+ T cell count. Kitaoka et al. reported that HIV-positive patients with complicated appendicitis had significantly lower CD4+ T cell counts than patients with uncomplicated appendicitis.^7)^ In this case, long-term inflammation following perforated appendicitis treated conservatively may have been exacerbated by HIV-associated immunosuppression, promoting chronic transmural inflammation and adhesion to the adjacent ileum. This mechanism is similar to the pathophysiology of fistula formation observed in Crohn’s disease and may have contributed to the development of the entero-appendiceal fistula.^8)^

Furthermore, although the possibility of a congenital communication cannot be entirely excluded, prior CT during the episode of appendicitis demonstrated a localized abscess between the appendix and small bowel, supporting an acquired etiology.

To our knowledge, previous reports have described communication between the appendix and ileum but have not documented direct intraluminal exposure of the appendiceal tip. In contrast, the present case clearly demonstrated appendiceal penetration into the ileal lumen on both macroscopic and microscopic examination. Continuous mechanical irritation by intestinal peristalsis and luminal contents may have caused mucosal ulceration, while concomitant apixaban therapy likely exacerbated the bleeding, resulting in overt gastrointestinal hemorrhage.

## CONCLUSIONS

This report described the first known case of an entero-appendiceal fistula identified during evaluation of overt gastrointestinal bleeding in a patient with a history of perforated appendicitis and HIV infection. This case highlights the importance of considering rare causes such as fistula formation, particularly in patients with a prior history of intra-abdominal inflammation and immunosuppression. Mechanical irritation of the protruding appendiceal tip, together with anticoagulant therapy, may explain the unusual presentation with overt gastrointestinal bleeding. Surgical resection was both diagnostic and therapeutic, and laparoscopic management was successfully implemented.
